# Barriers to mental health care and possible solutions in the young: *Yarns* with the Victorian Aboriginal community

**DOI:** 10.1177/00048674251384059

**Published:** 2025-11-05

**Authors:** Alasdair Vance, Janet McGaw, Naomi Tootell, Sandra Eades

**Affiliations:** 1 Academic Child Psychiatry Unit, The University of Melbourne and Aboriginal Mental Health Program/Developmental Neuropsychiatry, The Royal Children’s Hospital, Aboriginal Heritage, Alasdair Vance Matrilineal Northern Wathaurung Mt Emu People, Melbourne, VIC, Australia; 2 Faculty of Architecture, Building and Planning, The University of Melbourne, Married to Alasdair Vance, Northern Wathaurung Mt Emu people, Melbourne, VIC, Australia; 3 Faculty of Architecture, Building and Planning, The University of Melbourne, Melbourne, VIC, Australia; 4 School of Population and Global Health, Faculty of Medicine, Dentistry and Health Sciences, Sandra Eades is a Noongar Woman from Mount Barker, WA, Melbourne, VIC, Australia

**Keywords:** Australian Indigenous children and adolescents, barriers health care, yarns

## Abstract

**Objective:**

To identify the barriers accessing health (including mental health) services by Indigenous people in Victoria, Australia, and putative solutions, through *yarns* with 44 members of the Victorian Aboriginal community.

**Methods:**

This paper systematically explores grassroots barriers and potential solutions for Indigenous young people to engage and use health (including mental health) services. Elder-governed *yarns* were conducted via Zoom with 44 representative Victorian Aboriginal Elders, Healers, Senior and Junior people involved in the health and wellbeing of the Victorian Aboriginal community. These *yarns* were analyzed through an innovative, constructivist, multi-perspectival discursive grounded theory method.

**Results:**

Five pre-eminent themes emerged: the socio-economic barriers to services, the ongoing effects of colonization, disconnection and isolation from community and *Country*, pressures in society of living in two worlds and lack of cultural safety and racism. Detailed and rich day-to-day barriers and possible grassroots solutions were proffered.

**Conclusions:**

The analyzed *yarns* provide important detail about everyday barriers Indigenous peoples face in healthcare services and potential ways forward to improve the situation for Indigenous young people and their kinship networks. This paper can help shape future policy and its implementation. In particular, Aboriginal Community Controlled Health Organizations running traditional Indigenous healing programmes alongside Western health management, ensuring formal processes predominate and are monitored for their day-to-day effectiveness.

## Introduction

Indigenous people in Australia continue to suffer marked disadvantages in health and wellbeing compared to non-Indigenous people, despite 17 years of policy intended to close the gap. The most recent report on the National Agreement on Closing the Gap found that none of the Priority Reform areas were on track (Australian Government Productivity Commission, 2025). In fact, four of the socio-economic outcome areas had actually gone backwards (Australian Government Productivity Commission, 2025). The Australian Institute of Health and Welfare (AHIW, 2024a) has found that 30% of the health gap is explained by risk factors due to lifestyle, 35% is due to socio-economic factors (AIHW, 2024a), but 35% of the health gap remains unexplained by the available data (AIHW, 2024a). Lifestyle factors include inactivity, poor diet, alcohol consumption and obesity. In the period from 2022 to 2023, 34% of Indigenous people over the age of 15 consumed in excess of the National alcohol guidelines, 29% were daily smokers and 68% were overweight or obese (Australian Bureau of Statistics [ABS], 2024). Socio-economic factors include lower employment, lower income and poorer quality housing on average compared to non-Indigenous Australians. Only 56% of Indigenous people are employed compared to 78% of non-Indigenous Australians, 35% had incomes in the bottom 20% nationally and 33% lived in housing with major structural problems (up to 50% in remote areas) (AIHW, 2024a). The AIHW (2024a) surmises that the unexplained gap in health for Indigenous Australians could be due to a lack of access to health care services, lack of cultural safety, racism and structural disadvantages, given findings of earlier studies (AIHW, 2023b). But more research is required to tease this out.

There is certainly evidence that spatial and economic factors play an important role in accessing health services. In 2018–2019, 34% of Indigenous people reported the cost of seeing a health practitioner was too great, and 33% were hampered by inadequate transport, too great a distance to travel and/or poor availability of health care services in area, too long a waitlist or service not available when needed (AIHW 2023a). Similarly, approximately twice as many homeless Indigenous compared to non-Indigenous people reported not accessing services for the same reasons (AIHW, 2024b). International and national research has found cross-cultural differences and outright racism, which are also contributing factors. In Canada, Nelson and Wilson (2018) found that 52% of urban Indigenous people were receiving substandard health care, experiencing excessively long waiting times for care and were the victims of racist and discriminatory acts while in the health care system. Wilkinson et al. (2022) outlined multiple systemic barriers of racism in Australian health services such as a lack of organizational accountability and buy-in, ineffective service structure, funding and education, undervaluing of Indigenous health and wellbeing knowledge and negative framing of cultural safety to diminish its implementation. Meanwhile, Jennings et al. (2013) identified service system barriers for remote Australian Indigenous communities having annual health checks, including time pressures on patients and staff, health service protocols being unclear for staff and staff themselves being unclear about their responsibilities. Similarly, the Government of NSW (2022) reported that Indigenous people experienced racism, disrespect, derogatory and degrading management, personal and institutional exclusion and/or mistreatment that made them feel unsafe in or unable to trust services that seemed unresponsive despite flying the Aboriginal and Torres Strait Islander flags and having Indigenous artwork on the walls.

The current National Agreement on Closing the Gap (2020) was developed in response to the excoriating findings of the 10-year review (Holland, 2018) of the earlier 2008 Council of Australian Governments’ (COAG) National Indigenous Reform Agreement (Council of Australian Governments, 2009). It had identified a complete failure to involve Indigenous people in the original strategy-development process. Its focus on measures of success, defined through a Western lens, led to a blindness towards underlying causes. While the current Closing the Gap Agreement (2020) commits to shared decision-making, transforming government organizations, building the Aboriginal Community Controlled sector and data sharing, there has been no measurement of any outcomes in these areas to date. Furthermore, the measures for success or failure continue to be socio-economic, despite extensive research that shows the importance of cultural determinants of health for Indigenous peoples around the world (AIHW, 2023b; Country, 2019; De Leeuw, 2015).

Consequently, this research project seeks to interrogate the unexplained component of the health gap. It begins with a commitment to centering Indigenous world views, led by members of the Victorian Indigenous community, for the Victorian Indigenous community. We ask is improving access to health care services enough to promote better health and wellbeing? Or are there cultural determinants of health and wellbeing that Western health care services will never adequately provide? Indigenous people living in the south-east of Australia where the impacts of colonization early in the settler-colonial period were particularly severe, have particular barriers to health. The team wondered what solutions would emerge. This paper presents findings from a community needs *yarns* conducted with 44 participants, all interested in the relationship between culture and health, most also working in fields that are focused on supporting better health and wellbeing across Aboriginal Victoria. A culturally safe and appropriate method of analysis was also used (McGaw and Vance, 2023). Its purpose was to identify the extent to which culture was important to health and wellbeing, and to develop, in response, better models of delivering mental health care for Indigenous young people.

## Methods

### Establishing Indigenous leadership following cultural protocols

The study is co-led by an Indigenous child and adolescent psychiatrist, consulting with an Aboriginal health liaison unit within a major paediatric hospital in an Australian capital city that provides a cultural context. This study has developed from accepted socio-cultural protocols and is overseen by a Board of Elders and Senior People. Elders have a central and core role in the Aboriginal community (Busija et al., 2020) and their governance of cultural projects is consistent with how Indigenous communities work, nationally and internationally (Flicker et al., 2015; Kennedy et al., 2022). An Advisory Group of experienced Indigenous health professionals was established to provide additional guidance on navigating Western health care systems while maintaining cultural authenticity. The project was approved by the Royal Children’s Hospital Human Ethics Committee (2019.207/56941).

The process of seeking Elders’ guidance was long and involved. After several years of conversation between the project leaders (joint first authors) a group of six Elders and Senior People – 4 women, 2 men – from different regions around the state of Victoria consolidated by 2019. Membership has changed over the course of the project due to deaths and retirement, but Elders continue to guide the project leads in cultural matters as the study nears its conclusion. Quarterly board meetings have been supplemented with ad hoc lunches and phone *yarns*. An Advisory Group of Indigenous health professionals also provided important counsel, particularly in the early stages. A more detailed description of the Methods is provided in other papers (McGaw and Vance, 2023; Vance et al., 2024a, 2024b).

### Sampling and recruitment of community members

Representative Victorian Aboriginal community members – elders, healers, senior and junior people working in the health and wellbeing field – were identified by the elders as members of the Victorian Aboriginal community from varied walks of life and experience who could provide important insights into the relationship between culture and health. They included people of varied ages, genders, living in Victoria across a range of locations (urban and rural) and inclusive of 31 traditional language groups (see Table 1). Participants were consented by an Indigenous research assistant, who also conducted the *yarns.* Forty-six individuals participated. Two participants subsequently withdrew from the study.

**Table 1.** Participant characteristics for the 44 community needs assessment *Yarns*.

	Northern VIC	Southern VIC	Eastern VIC	Western VIC
Males, females	8,9	4,5	4,5	4,5
Urban, rural	9,8	3,6	5,4	4,5
E/H/SP/JP	6,3,5,3	4,1,3,1	4,1,3,1	4,1,3,1
Tribe	17	22	20	21

Northern/Southern/Eastern/Western Vic = region of Victoria, Australia; urban = metropolitan living, rural = country town or farm property living; E/H/SP/JP = elder/healer/senior person/junior person working in health and wellbeing field in Aboriginal Victoria; Tribe = tribal affiliation of participants (commonly multiple groups).

### Yarning with community through COVID-19

A hybrid method of Community Participatory Action Research (CPAR) and Grounded theory was employed as the overarching research methodology for the *yarns*, to manage the inherent Western bias implicit in most research methods. Grounded theory is a well-defined, methodologically rigorous qualitative research methodology that has been used in conjunction with CPAR by other scholars working with Indigenous communities (Bainbridge et al., 2013; McGaw and Vance, 2023; Murrup-Stewart et al., 2021; Priest et al., 2017). Grounded theory commences with open questions, rather than a hypothesis or theory, focuses on social actions and processes and uses an inductive process to generate theoretical abstractions arising from the participants’ data (Charmaz, 2006; Glaser, 1992). CPAR, meanwhile, privileges Indigenous ways of knowing, doing and being and ensures community is at the centre of the research (Kovach, 2009; Quinn, 2022). Indigenous ways of knowing, doing and being are rooted in an entirely different epistemology, ontology and axiology to Western health care. Knowledge is situated within a network of human, *Ancestral*, geographic and *Totemic* relationships that are not quantifiable, not measurable and not individually defined, but passed on by ‘mob’ (Aboriginal community defined by connection and belonging to each other and *Country*) through ‘*Storying*’. The method for the community consultation was *yarning*, which is an Indigenous method of making sense of lived experience with another (Geia et al., 2013). It is a specific, cultural, fluid and dynamic process and method of knowledge exchange that is culturally safe (Kennedy et al., 2022; Shay, 2021): so sensitive issues and intimate information can emerge through two-way knowledge sharing and so all involved are enriched by the experience. Involving a well-known Indigenous community member to lead the *yarns* was crucial given the known strong insider versus outsider status of Indigenous community membership (Innes, 2009; Merriam et al., 2001). The reciprocal kinship connections, obligations and responsibilities they shared aided the *yarning* process and smoothed the way (Dew et al., 2019).

The *yarns* were guided by four open-ended questions that were circular unfolding narrative journeys: What does culture mean for you and your mob? What cultural practices aid and/or maintain health and wellbeing for you and your mob? Who best governs cultural practices in your mob? What are practical issues for enabling culture to be central to mental health care? Because of COVID-19 movement restrictions, the team adopted the format of one-on-one ‘zoom*-yarns’* between an Indigenous research assistant and a representative sample of elders, healers, senior and junior people, involved in health and wellbeing across the Aboriginal nations in the Victorian Indigenous community (see Table 1). While the social disruption of COVID-19 to the community and project was the primary reason for the change, the method also had its strengths: the zoom-*yarns* captured a broader variety of perspectives – young and old, working in health and independent community members; they ensured the quiet voices were heard; they were deeply reflective – in fact, they seemed to be a source of solace amid long durations of social isolation; they were substantial – 45–60 minutes, or 8–10 pages of transcribed conversation on average; and they were easy to record and transcribe.

### Analyzing the community yarns to co-design an adjuvant therapy

The zoom-*yarns* were analyzed using a multi-perspectival, discursive, constructivist approach to ensure the participants’ experiences drove the findings (Charmaz, 2006; Glaser, 1992; McGaw and Vance, 2023). Human ‘coders’ inevitably bring a particular lens: social, cultural, educational and disciplinary. To reveal the blind spots that might emerge from the intrinsic interests and disciplinary training of a single coder, a multi-perspectival, discursive approach was adopted. Four coders reviewed all the *yarn* footage and/or transcripts. Each had a distinct social, cultural and disciplinary perspective: a former AHLO without tertiary training, mental health professional (psychiatry), social scientist (anthropology) who had worked for many years gathering community stories for Native Title, and the spatial disciplines (architecture and cultural geography) who had worked for many years with the Victorian Aboriginal community on place-making projects. One of the coders was the research assistant who conducted the zoom-*yarns* – a participant-researcher. Niklas Luhrmann’s (1986) autopoietic systems theory observes that group processes like this allow for the spontaneous emergence of new ideas, a key ambition of grounded theory.

Each team member worked independently at first to watch/read all the *yarns* and complete initial coding and provisional thematic coding (Charmaz, 2006; Glaser, 1992). Memos were written at every stage to enable the participants’ experiences to be explored as the coders critically self-reflected on their own pre-existing ideas and assumptions (Cooper and Burnett, 2006). Discursive reflexivity ensured the coder remained mindful of their positionality. Once complete, the team met, also in *Zoom*, over four extended sessions, where each coder took their turn to share what they had ‘discovered’ through each *yarn*. Sometimes the group was united in what they believed had emerged from the data. But, at times, the dissonance between perspectives was the key finding. Mouffe (1999) coined the term ‘agonistic pluralism’ to describe a process of ‘struggling with’ difference. Consensus, she contends, simply reinforces a dominant discourse. Through multi-perspectival discourse, all views were equally valued and included in the analysis of the *yarns*.

## Results

The yarns coalesced and focused on four primary concerns: definitions of culture; ways of sharing and practicing culture; ways it is perceived to maintain health; and barriers to accessing health care – Western and culture-centred – and the impacts on health and wellbeing. This paper focuses on the latter concern: barriers. Within this theme, there were five themes: the socio-economic barriers to services, the ongoing effects of colonization, disconnection and isolation from community and *Country*, lack of cultural safety and racism and pressures in society of living in two worlds. Possible solutions were also shared in detail.

### Socio-economic obstacles to health care

Predictably, many of the participants affirmed that socio-economic disadvantage presented a considerable barrier to accessing health services. Despite the promise of Universal healthcare in Australia, medical care can be costly. Location played a part. Many described inadequate and unreliable transport and the unavailability of health services in the area, which meant long distances to travel. When they presented, there were often long waitlists and/or a lack of the type of services they needed. Many disliked their local mainstream health service and/or were afraid to present because of feelings of social exclusion or racism so would choose to travel elsewhere or not go at all if it added too much difficulty. The never-ending struggle to make ends meet financially was a widespread concern. All the participants promoted equity and self-determination. They wanted health care to be properly funded, so the lack of money is not an obstacle to health service provision.

‘Australia should pay the rent of illegally squatting here last 200 odd years. Yeah – equal share so we can have plenty too’ (Participant 32)

‘Money to have a healthy place, no mold on the walls and no mice in the kitchen. Able to travel where and when I need to rather than waiting for paycheck every month’ (Participant 6)

But solving the socio-economic barriers seemed to be a complex challenge and was not considered to be sufficient on its own. There were deeper structural barriers that perpetuated Western medical services as alienating environments.

### Ongoing effects of colonization

The ongoing effects of colonization were noted by almost every participant as a key barrier to equitable access to healthcare services. Aboriginal Victorians have a long history of negative experiences with settler-colonial authorities, and the public healthcare system is continuous with this. For example, Aboriginal Victorians were historically not allowed inside hospitals and could be treated only on hospital verandahs, if at all. Many Aboriginal Victorians were also separated from their families as children by settler-colonial authorities purely based on race – a practice that only ceased in the 1960s. As a result, many Aboriginal Victorians have been taught by their parents, grandparents and great grandparents never to trust settler-colonial authorities. These historical issues and others like them were repeatedly raised by *yarning* participants to explain why many Aboriginal Victorians continue to avoid accessing healthcare services today. These issues are also well known within the existing literature.

However, many *yarning* participants also highlighted the colonial tactics healthcare service providers in Victoria continue to deploy today as a way of maintaining power and control over Aboriginal people, all the while claiming allegiance to the principles of ‘self-determination’. For example, non-Indigenous powerbrokers often ensure that outspoken, non-compliant Indigenous staff members do not gain power, influence or control. They do this by not renewing certain people’s contracts, e.g., or by denying a person a continuing position:

‘Blackfellas who speak up, tell the truth, have a target on their back. They always put quiet ones in who do what they’re told’. (Participant 27)

Another strategy commonly referred to by *yarning* participants involved separating Indigenous staff along supervision, governance and/or reporting lines, thus preventing the formation of a cohesive Indigenous staff group that could potentially support greater self-determination. Instead, Indigenous staff often find themselves working alone (i.e. without Indigenous colleagues) across multiple health organization service departments. Importantly, these moves are not interpreted by *yarning* participants as careless or accidental, but rather as part of a deliberate colonial strategy:

‘They put on an Indigenous mental health staff worker and made sure she was supervised by a white person so they kept our mob separated. They divide and rule because they are afraid of self-determination’. (Participant 15)

*Yarning* participants also pointed to the delay tactics healthcare services use to reproduce colonization and de-prioritize Indigenous issues. One yarning participant, e.g., narrated the story of a health organization that affirmed its support for Indigenous reforms framed by government policy yet repeatedly left the reforms off business meeting agendas. Later, after relevant changes had been agreed upon, management claimed that budget lines were insufficient to implement them. Later still, they employed outside consultants who subsequently quashed reforms on the grounds of cost, a view with which management quietly agreed:

‘The masterplan included an Indigenous healing garden, but city suits came in and said much cheaper to put in introduced plants so Executive dropped the idea’. (Participant 42)

What these examples suggest is that *yarning* participants consider the problems of racism and discrimination within healthcare organizations not as problems of the past, but as problems that persist in the present. While their forms are more subtle than they were historically, they thwart progress and self-determination even while healthcare services proudly purport to be doing the opposite. These issues are known not only by Aboriginal healthcare workers, who represented many of the *yarning* participants, but also by the communities they serve and in which they live, and they help explain why Aboriginal Victorians do not trust healthcare services today.

A host of practical solutions were canvassed by the participants: The benefits of Aboriginal Community Controlled Health Organizations (ACCHOs) as places of cultural safety featured prominently. These are places associated with self-determination. Prioritizing Indigenous knowledge of health and wellbeing, building trusting and respectful relationships and monitoring organizations to ensure they demonstrate buy-in and are accountable were also identified as important practices. But, on the whole, both mainstream and Aboriginal health service providers still prioritized a Western model of medical care that many participants considered to be inadequate on its own. Health is a holistic construct in Indigenous culture. People need emotional and spiritual nurture as well as physical and mental care. And importantly, *Country* needs to be healthy too, for people to be healthy.

### Disconnection from community and Country

The vast majority of participants described in detail periods of their lives when they experienced the desolation of being separated from their community and/or their *Country*. Often this was linked with limited or no opportunities to practice Culture and immerse themselves in health-enabling practices. This is a consequence of settler-colonial policies in Victoria dating back to 1869 when the first Aboriginal Protection Act was enacted, legalizing (under Western law) the removal of Indigenous people to reserves and missions, disconnecting people from their *Country* and cultural practices. The Amendment to the Act in 1886, which allowed for the removal of children from their families, disrupted kinship ties. Lasting intergenerational trauma from this disconnection was extensively discussed. In particular, sadness about being denied knowing and learning more about their Spiritual origins associated with *Creator Spirits* in particular *Country* and/or *Totemic Spirits* was frequently raised. Interestingly, stories of substance abuse and criminal behaviour seemed to accompany such periods of desolation.

‘Being unwell–It’s a disconnect – not knowing who you are where you are from what your lines are Spiritually – it is really stressful and you are alone in it all on your own – no self-esteem, no confidence, an outcast no belonging nothing there empty and then being traumatized becomes your culture all enmeshed up and a mess’ (Participant 16)

‘I get so home sick for my *Country* my *Place* – miss painting with my mob where I belong, being connected up again rather than adrift. People there have known me since I was a kid. The weight is there off *Country* – definitely’. (Participant 19)

Almost every participant mentioned in varying degrees of detail connecting up with community and *Country* being the answer for the desolation of lost cultural identity and social isolation. Many participants talked about meaningful connections when no words are spoken, but so much is communicated through deep listening and non-verbal cues. Simply being together enables reciprocal understandings to be shared and developed collectively.

‘Being accepted. I was very valued. I knew how to navigate. I still felt safe within my own community. But I was feeling I had a sense of belonging. You know where the pot is, you know where the kettle is, you know where to go and feed yourself. Always knowing I could maintain that connection even if I worked in government, I always made time to drop in and re-energise myself’. (Participant 4)

‘A young person who can’t speak to family – it is really important they have someone to really and carefully listen to them who is safe and trustworthy and consistent for them–it makes the biggest impact on someone’s life’. (Participant 1)

‘I definitely feel a sense of clear calm and acceptance on my *Country*, but I don’t feel unaccepted here when I’m off *Country*. I think over time increasingly knowing who you are as an individual, as a sister, an Aunty, a grandmother, you get a sense of who you are that allows you to belong wherever you are. Because you practice Culture according to the protocols’. (Participant 23)

Importantly, many emphasized connecting with *Country* being central to true and ongoing mental health and wellbeing: For many, this was infrequent as they, or their parents or grandparents before them, had moved to the city for work. Returning to *Country* was described viscerally and powerfully. Others described seeking out bushlands near where they lived so they could feel the earth, hear the birdsong, and block out the hubbub and traffic of urban life.

‘Being on *Country*–It’s a pretty special feeling when I’m on my *Country*. I feel complete, I feel connected, like I belong, strong within myself, like I can take on the world really’ (Participant 2).

### Racism and lack of cultural safety

Racism and lack of cultural safety were on a continuum and ever-present in Western health care settings. There were numerous examples of insidious racism: Being served last when presenting to health services, being told no appointments available, being asked for identification when being triaged while others are allowed in before them, being made to wait even when surgery was needed, medical staff appearing to be disgusted by their appearance and smell, and refusing to touch and examine them thoroughly.

Racism is still the biggest problem and obstacle. Both overt and subtle is still so strong in our lives. Community needs to support each other. The government is still about divide and conquer that they’ve been doing since colonisation. It’s about separation and all those things. (Participant 7)

But even in Western health services that had the hallmarks of cultural safety, participants overwhelmingly felt that they were not providing culture-informed care, despite Aboriginal art or flags being on the walls or footprints being embedded in the floor. This included the intake, assessment, diagnosis, formulation, management and discharge processes. Many participants were distrustful and dismissive of Western medication and/or psychotherapy options provided in the mental health field. Rather, they emphasized how powerful and effective traditional Indigenous practices were.

‘No. I don’t think the mental health system caters for our mob at all. If you think about the white ways, they do assessments but none of them ever cater for culture. Very, very rare that they cater for culture. Treatments are ‘white’, usually some form of medication which dulls your senses and your feelings and makes you a bit of a zombie for however long. Culture has the opposite effect. I’ve noticed her and watch her, and when she gets in with clapsticks, there is none of that fidgety, fidgety. When we do Culture, I don’t see it because it’s not there. She’s calm for those times’. (Participant 3)

‘Mental Health models are Western centric, about individuals and they don’t understand collective healing practices – I discovered mindfulness, that I make as Koori as possible. So I try to make it more specific to me and my family’. (Participant 42)

Few participants provided any clear advice about managing racist interpersonal or institutional experiences aside from ignoring them and trying to move on with their day and not be defeated by them. However, most participants talked about the importance of internal reminders of their cultural identity as an Indigenous person and regularly connecting with community and *Country*.

Despite this, very few of the participants advocated *only* providing traditional Indigenous cultural practices in health services. Rather, the majority affirmed that both Western and Indigenous health and wellbeing approaches should be offered without either being watered down by the other. The model with the most support was having a comprehensive Western health assessment and management offered alongside and nested within ACCHOs that also provide traditional Indigenous cultural healing.

‘I think the other thing is, Western knowledge of mental health, then you have a Cultural knowledge of healing and ways of being, they need to be held equally. It shouldn’t be either or. If you put them together they are both useful’. (Participant 13)

### Pressure of living in two worlds

Many participants outlined how difficult it is to live in Western society, work, manage a household while also engaging in Aboriginal cultural ways and supporting your family and wider kinship group. Settler culture is omnipresent. It is very hard to understand, learn, practice and internalize Aboriginal cultural ways as well.

‘Too much multi-tasking trying to be Aboriginal and also Western–there is too much focus on multi-tasking in the Western world and it’s really bad for your brain to do too much multi-tasking. It burns people out’. (Participant 31)

‘Distressing mainstream mental health way is the dominant way–I feel like Cultural Practices are a much better fit with our mob. We have two completely different ways of healing. For example, in mainstream they would advise counselling, but our way is going out on *Country*, being on the land, being with our mob, being with your people. Two totally different worlds’. (Participant 18)

The clear majority of participants shared that they sought out a time to live in their *Country* for a period of at least once in their lives. While many were drawn back into the city, they retained an aim of living a more profound, centred and slow-paced existence in their *Country* again. Older participants said that the community needed to be mindful of young people – including those in out-of-home care – to make space for them to join with the community in sharing the delight of being connected again with *Country*.

‘Every day we must remember to look after ourselves – de-stress and not be so busy busy – go out of the rat race and go home to *Country*. Take the child out of the toxic Western environment so they can connect to land, water and community and try different Cultural things. Even harder for mob that are removed from family as don’t have that option – we have to make an option for them’. (Participant 12)

## Discussion

There is much revealed in the *yarns* that is consistent with the extant national and international literature (AIHW, 2023a; Beyond Blue, 2024; Nelson and Wilson, 2018; Wilkinson et al., 2022): Indigenous people in south-east Australia grapple with significant and ongoing financial pressures that could be remedied by genuine resource equity and implemented self-determination. Despite the high population density compared to other regions of Australia, they also find Western health services hard to access, frequently unavailable and believe they are regularly excluded from utilizing them. The most common proposed solution is for ACCHOs to be better funded to provide comprehensive traditional Elder-governed Indigenous Cultural practices alongside but separate from Western health management (Asamoah et al., 2023; Beks et al., 2023; Conway et al., 2017; Davy et al., 2016; NACCHO/RACGP, 2019). ACCHOs are seen as central for powerful and effective Indigenous knowledges of health and wellbeing to be of primary health care importance, respectful and trusting relationships in health services to develop and be maintained, and for culturally appropriate monitoring of organizations to occur.

Most of the literature and policy documents focus on improving health care facilities and access to them. This includes strategies such as better coordination of service systems and building trusting clinical relationships through clear respectful communication (McBain-Rigg and Veitch, 2011; Nolan-Isles et al., 2021), better intersectoral collaboration (Nguyen et al., 2020; Wilkinson et al., 2022) and cultural training and ongoing audits of the effectiveness of feedback systems (Jennings et al., 2013). While these are important to our participants, they emphasized the equal importance of connecting to culture, community and *Country* to be healthy. Our participants felt living off *Country* was usually linked to states of demoralization, inner emptiness and a deep yearning or longing, akin to being separated from a life partner. Demoralization, comprising a loss of hope, meaning, purpose and existential distress, is a parlous state most frequently linked with palliative care for cancer sufferers in Western health (Robinson et al., 2016). Holistic connection to community and *Country* and living moment by moment in a cultural way, practicing traditional healing skills such as deep listening, are also solutions, as other Aboriginal researchers have noted (Calma et al., 2017; Dudgeon et al., 2016). The exponential increase in daily pressures facing Indigenous people living in Western and Indigenous worlds is a serious and relentless barrier to health and wellbeing: constantly having to understand, learn, practice and internalize Western and Indigenous cultures given their alien epistemological, ontological and axiological bases (McGaw and Vance, 2023). Overwhelmingly, Indigenous Victorians seek opportunities to connect with community and Country to experience their peace, balance, centredness and healing power (Vance et al., 2023, 2024b). Tellingly, disconnection from these frequently leads to decreased practice and knowledge of cultural ways of healing and being well. If possible, being in rural bush settings as much as life allows, and especially being on *Country*, affords a slower paced, profound and centred healing lifestyle to flourish (Vance et al., 2023, 2024a).

Current policy now supports practices to enhance cultural safety within health care organizations. The Victorian Department of Health (2023) proposes a comprehensive and detailed map to includes developing ‘shared meanings, knowledge and respect’, removing ‘unconscious bias, racism and discrimination’, supporting ‘identity and experience’ while minimizing ‘denial’, and sharing ‘decision making, governance and financial resources’. The Australian Government Department of Health (AGDoH) (2019) focuses on employment of Indigenous staff in health services and engaging with the Indigenous community to tackle language and communication issues, Amery, 2017; Australian Digital Health Agency (ADHA)/Queensland Aboriginal and Islander Health Council (QAIHC), 2023; Queensland Health, 2015. Beyond Blue (2024) called for recognition of the centrality of holistic social, cultural, economic and legal connections at community and Country. But these policies belied the everyday experiences of many participants. They repeatedly outlined stories of the underhand duplicity of health professionals maintaining their power, influence and control through saying one thing (e.g. self-determination) but doing another (e.g. racist discrimination, harassment and/or exclusion). They were most scathing about tokenistic ‘window dressing’ – Indigenous artworks on the walls – if it was juxtaposed with an experience of marginalization and exclusion. This has also been recognized by other researchers (McBain-Rigg and Veitch, 2011). Where this occurred, distrust of the Western health management offered was exacerbated.

Participants who worked in Western health care organizations noted that informal processes trumped formal organizational processes. This can be understood as an example of the ‘private’ self being privileged over the ‘public’ self (Johnson and Majewska, 2022; Tedeschi, 1986). Tedeschi (1986) defined the former as comprising the essential true beliefs, attitudes, coping styles and emotional and behavioural patterns of the person, while the latter consists of the desired public projections of these attributes. Often the ‘private’ self is associated with informal processes that are a main driver of colonizing racism. Informal processes are unstructured, unintended, occur anywhere and usually involve procedural learning, i.e., ‘how things really work and decisions are really made’ (Johnson and Majewska, 2022). They are invariably more important to how health services actually function than the formal processes often monitored as part of an organization’s function (Johnson and Majewska, 2022). Lea (2020) provided a perspicacious analysis of these informal processes in her excoriating dissection of the Federal Government of Australia’s 2007 Northern Territory Intervention targeting Indigenous communities. Accordingly, to optimize health service functions for Indigenous peoples, formal processes within a project-based organizational model need to predominate and be monitored for their day-to-day effectiveness (Hobday, 2000). Remembering their Indigenous identity, connecting to community and *Country*, and practicing culture was seen by participants as an important resilience factor, being strong culturally strengthened participants against the effects of colonization.

The main limitation of this project is that we only *yarned* with 44 Victorian Aboriginal community members. They represented a diverse group and many had significant experiences working in the Aboriginal community health sector. They were therefore able to speak from both their own experiences of accessing health services as well as the experiences of those they live and work with. We were unable to yarn with more members of the Victorian Aboriginal community due to COVID restrictions and budgetary constraints. However, future community consultation through *yarning* could address this weakness. Strengths of the study included its leadership by an Indigenous researcher and clinician; governance by Elders; support of an Aboriginal Advisory Group; and consultation through *yarning* with a representative sample of elders, healers, senior and junior people from all regions of Victoria, Australia who have an interest in culture and health and wellbeing. Meanwhile, the size of the sample and sophisticated and careful analytic strategy used to explore the themes from the *yarns* is recognizable as valid, reliable and generalizable to Western qualitative researchers (McGaw and Vance, 2023; Vance et al., 2024a, 2024b).

## Conclusion

In conclusion, the analyzed *yarns* provide rich detail about everyday barriers Aboriginal and Torres Strait Islander young people and their kinship networks face in healthcare services and ways forward to improve health. There are ample data from the community *yarns* to enrich the form and content of holistic, functional and strengthened healthcare programmes. The five key themes that emerged were the socio-economic barriers to services, the ongoing effects of colonization, disconnection and isolation from community and *Country*, lack of cultural safety and racism and pressures in the society of living in two worlds. The data from this study are relevant for Indigenous people living in the south-east of Australia, where colonization has arguably had the most long-standing and deleterious impact (Sherwood, 2013). But these data are also pertinent for all places subject to cultural dispossession and dislocation. This paper can help shape future policy and its implementation: especially ACCHOs running traditional Indigenous healing programmes alongside Western health management, ensuring formal processes within a project-based organizational model predominate and are monitored for their day-to-day effectiveness (Hobday, 2000).
